# Performance of 5-aminolevulinic-acid-based photodynamic diagnosis for radical prostatectomy

**DOI:** 10.1186/s12894-015-0073-y

**Published:** 2015-08-01

**Authors:** Hideo Fukuhara, Keiji Inoue, Atsushi Kurabayashi, Mutsuo Furihata, Taro Shuin

**Affiliations:** Department of Urology, Kochi Medical School, Kohasu, Oko, Nankoku, Kochi 783-8505 Japan; Department of Pathology, Kochi Medical School, Kohasu, Oko, Nankoku, Kochi 783-8505 Japan

## Abstract

**Background:**

The aim of this study was to investigate whether we could detect positive surgical margins during open and laparoscopic radical prostatectomy by 5-aminolevulinic acid (ALA) photodynamic diagnosis (PDD) and mapping of red fluorescence in human prostate cancer cells.

**Methods:**

All 52 patients were diagnosed with prostate cancer by biopsy. They had a positive core in the apex or highly suspicious positive margins. Open and laparoscopic radical prostatectomy was performed in 18 and 34 cases, respectively. One gram of ALA solution was given intraoperatively, orally through a stomach tube. An endoscopic PDD system, including a D-Light C, CCU Tricam SLII/3CCD CH Tricam-P PDD, and HOPKINS II Straight Forward Telescope 0°, was used. The D-Light C light source was equipped with a band-pass filter. The CCU Tricam SLII/3CCD CHTricam-P PDD video camera system was equipped with a long-pass filter. The laparoscopy optic component was equipped with a yellow long-pass filter.

**Results:**

One of the 52 patients had a red-fluorescent-positive margin of the excised whole prostate and the positive surgical margin was histologically confirmed. In the section of excised prostate, we obtained 141 biopsied samples. The sensitivity and specificity were 75.0 % and 87.3 %, respectively.

**Conclusions:**

Intraoperative ALA-PDD is feasible. However, heat degeneration and length of positive surgical margin have crucial influences on red fluorescence. In future, a randomized clinical trial should be carried out.

## Background

The number of new prostate cancer cases in Japan has shown a consistent increase due to widespread acceptance of prostate-specific antigen (PSA) mass screening. The number of prostate cancer cases in Japan reached ~42,000 in 2006 [1]. Radical prostatectomy for localized prostate cancer is a highly effective standard treatment modality. However, the rate of positive surgical margins for radical prostatectomy was reported to be 14–26 % [2–5]. Positive surgical margins in prostate cancer are a significant risk factor for biochemical recurrence and lead to unfavorable prognosis [6–9].

5-Aminolevulinic acid (ALA) is a naturally occurring metabolite that is a precursor of porphyrin in heme biosynthesis. Exogenous ALA leads to accumulation of the potent photosensitizer protoporphyrin (Pp)IX in mitochondria. PpIX is known to accumulate more in malignant and proliferating tissues than in normal tissues [10–14]. In this way, PpIX selectively accumulates at a significant level in tumor cells. PpIX is an effective photosensitizer and fluorescent substance in heme biosynthesis.

5-ALA-mediated photodynamic diagnosis (PDD) is used widely in various cancers, including bladder cancer. If we could adapt this technique to PDD during radical prostatectomy, we could improve the rate of positive surgical margins. Zaak et al. and Adam et al. reported the feasibility of intraoperative ALA-PDD for the detection of positive surgical margins [15, 16].

In this study, we focused on two aspects. The first was to investigate the feasibility of intraoperative ALA-PDD for the detection of positive surgical margins. The second was to demonstrate the predominant accumulation of PpIX in human prostate cancer cells compared to normal prostate cells by the use of ALA-PDD on the divided surface of excised prostate. Thus, we investigated the feasibility of intraoperative PDD using 5-ALA in prostate cancer.

## Methods

### Patients

Intraoperative ALA-PDD with radical prostatectomy was approved by the Ethical Committee of Kochi Medical School, Japan in January 2008. We enrolled 52 patients with histologically confirmed adenocarcinoma-type prostate cancer in the Department of Urology, Kochi Medical School Hospital between February 2009 and August 2012. All patients were informed about the potential efficacy and adverse events, such as skin photosensitivity, transient elevation of serum aspartate aminotransferase (AST) and alanine aminotransferase (ALT), nausea, and vomiting. All patients gave written informed consent. The 52 patients had a median age of 67.1 years (range, 56–76 years), initial PSA level of 7.71 ng/ml (range, 0.008–76 ng/ml), and histologically confirmed adenocarcinoma of the prostate apex (positive core rate 31.4 %; range, 8.3–75 %), according to the general rules for clinical and histological studies on prostate cancer, or an expected ≥25 % probability of extraprostatic extension (25.9 %; range, 3–59 %) defined by the Japan PC table (Preoperative nomogram developed for clinically localized prostate cancer in Japan) [17]. Patients were stratified according to the D’Amico classification into low-, intermediate- and high-risk groups. Twenty-eight patients were categorized as low risk, 13 as intermediate risk, and 11 as high risk. All 52 patients underwent radical prostatectomy; 10 received preoperative deprivation therapy; 34 underwent endoscopic retroperitoneal radical prostatectomy; and 18 underwent open retroperitoneal radical prostatectomy according to the technique described by Walsh [18]. The patient characteristics are shown in Table 1.

**Table 1:** Patients’ characteristics

Object	2009/2–2012/9	
	52cases of prostate with operation	
	(Open: 18 cases, Laparoscopic: 34 cases)	
Age: average (range) (years old)	67.1 (56–76)	
Initial PSA (range) (ng/ml)	9.76 (3.07–86.69)	
Pre-op PSA (range) (ng/ml)	7.71 (0.008–42.57)	
Pre-op MAB (+/−) (cases)	10/42	
Apex (biopsy) (+/−) (cases)	17/35	
Probability of EPE (%)	25.9 (3–59)	
in Japan PC table		
Clinical stage (cases)	cT1c	34
	2a	9
	2b	7
	3a	2
	N, M	0
Gleason score	2–6	30
in biopsy specimen (cases)	7	12
	8–10	10

### Administration of 5-ALA

We administered ALA as a photosensitizer for ALA-PDD. ALA hydrochloride (COSMO BIO, Tokyo, Japan) was dissolved in 50 ml 5 % glucose solution. One gram of ALA solution was given intraoperatively, orally through a stomach tube.

### System of PDD

An endoscopic PDD system (Karl Storz, Tuttlingen, Germany), including a D-Light C, CCU Tricam SLII/3CCD CH Tricam-P PDD, and HOPKINS II Straight Forward Telescope 0°, was used. The light source, D-Light C (300 W xenon arc lamp), was equipped with a band-pass filter designed to transmit blue light (excitation wavelength, 375–445 nm; for excitation of fluorescence). The video camera system, CCU Tricam SLII/3CCD CHTricam-P PDD, was equipped with a long-pass filter designed to block blue light (for observation of fluorescence; fluorescence emission wavelength, 600–740 nm). The laparoscopy optic component was equipped with a yellow long-pass filter to reduce blue excitation light and enhance the fluorescence color contrast. The light source can be instantly switched between white light mode for conventional observation and blue light mode for fluorescence handled by a camera controller.

### Intraoperative procedure

In open radical prostatectomy, we placed the PDD laparoscope into the surgical field under general anesthesia and observed the surgical margins using the white and blue light modes. Blue light illuminated the surgical margins of the urethral, bladder, and rectal side, and enabled observation of the status of red fluorescence in the darkened operating room. To avoid unnecessary tissue removal, only the fluorescence-positive region was biopsied, for ethical reasons. When the fluorescence-positive region was close to a surrounding organ, such as the urethral sphincter and rectum wall, the region was removed as completely as possible with attention being paid to functional preservation of the surrounding organ. We sampled red-fluorescence-positive specimens and compared them with the pathological results.

### Examination of excised prostate

Soon after harvesting the whole prostate, we also performed laparoscopic PDD to detect red-fluorescence-positive surgical margins (base, apex, lateral, and rectal side). If a red-fluorescence-positive area was observed in the surgical margin of excised whole prostate, this area was biopsied by cold-cup for pathological examination. Subsequently, we divided the excised prostate in the median direction. The divided surface of excised prostate was also subjected to PDD. We separated the divided surface into four areas, and one specimen that was fluorescence-positive or -negative was biopsied by cold-cup in each area. The fluorescence intensity in each specimen was roughly divided into four categories (none, weak, moderate and strong), which was previously used in a clinical study on brain tumors performed by Miyoshi et al. [19]. The histological results were determined by two pathological specialists without knowledge of the results of fluorescence intensity.

### Statistical analysis

The diagnostic accuracy of the divided surface in excised prostate was calculated on the basis of comparison between fluorescence intensity and pathological results according to general rules for clinical and pathological studies on prostate cancer.

## Results

### PDD inside surgical margin

All 52 patients underwent retropubic radical prostatectomy and PDD. Eighteen patients underwent open radical prostatectomy and 34 underwent laparoscopic radical prostatectomy. There were no red-fluorescence-positive and pathologically positive surgical margins inside the surgical margin in either the laparoscopic or open surgery group.

### PDD in excised whole prostate

**Fig. 1:**
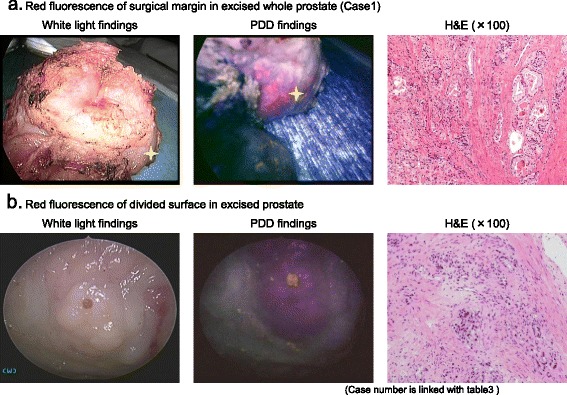
PDD finding in surgical margin and divided surface of excised prostate. **a**. Fluorescence-positive area of excised whole prostate was detected at the surgical margin in the base side (Gleason score 6). **b**. Longitudinal section in divided surface of excised prostate showed fluorescence-positive area in the transitional zone (Gleason score 7)

**Fig. 2:**
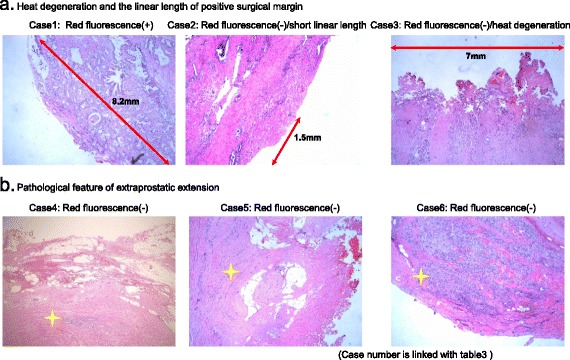
Association between pathological finding and red fluorescence in positive surgical margin and extraprostatic extension. **a**. In a case of heat degeneration and short linear length (<3 mm) of positive surgical margin, red fluorescence was not observed. **b**. All 3 cases of extraprostatic extension had no red fluorescence with tissue covering the tumor cells

Soon after harvesting the prostate, we performed PDD on the surgical margins of the excised whole prostate. One case (Case 1 in Table 2) demonstrated a red-fluorescence-positive surgical margin of the excised whole prostate on the basal side. Pathologically, this area was classified as Gleason score 6 adenocarcinoma (Fig. 1a). In this case with a red-fluorescence-positive surgical margin, the linear length of the positive surgical margin was 8.2 mm, without heat degeneration (Fig. 2a). However, another two cases had a pathological positive surgical margin with no red fluorescence. The first case showed heat degeneration of the surgical margin, with a 7-mm-long positive surgical margin (Case 3 in Fig. 2a, Table 2). In the second case, the positive surgical margin was only 1.5 mm long, without heat degeneration (Case 2 in Fig. 2a, Table 2). Three cases diagnosed as extraprostatic extension had no red fluorescence. In these cases, tumor cells were not present on the surface of the prostate. In fact, tumor cells were covered with prostatic capsule or peripheral fat tissue (Fig. 2b, Table 2). Ultimately, the rate of false negativity was 3.8 % in total. After examination of surgical margins, we divided the excised prostate from 29 cases and biopsied each area by cold-cup with PDD. We obtained 141 biopsied samples in all. Fluorescence positivity was found in 31 samples, while pathological positivity was found in 20 samples (Fig. 1b). The overall sensitivity and specificity were 75.0 % and 87.3 %, respectively (Table 3).

**Table 2:** Individual results of positive surgical margin and extraprostatic extension

No	Operation	MAB	Preop PSA (ng/ml)	Probability of EPE (%)	D’amico classification	pT stage	Gleason score	Fluorescence intensity	Margin status	Heat Degeneration (pathological)	Linear length of surgical margin (mm)
1	open	none	5.8	19	low	2b	4 + 5	moderate	positive surgical margin	-	8.2
2	open	none	42.57	27	high	3b	5 + 4	none	positive surgical margin	+	1.5
3	Laparoscopy	none	21.15	59	intermediate	3a	4 + 3	none	positive surgical margin	-	7.0
4	Laparoscopy	none	6.87	30	low	3b	3 + 4	none	Extraprostatic extension	-	1.2
5	Laparoscopy	none	11.93	21	high	3a	4 + 5	none	Extraprostatic extension	-	2.0
6	Laparoscopy	none	21.15	59	intermediate	3a	4 + 3	none	Extraprostatic extension	-	3.3

*MAB*, Maximum androgen blockade, *EPE*; Extraprostatic extension

**Table 3:** Diagnostic accuracy of PDD in excised whole prostate and divided surface of excised prostate

The surgical margin status of excised whole prostate	
Red fluorescence positive surgical margin	1
Pathologically positive surgical margin	3
Pathologically extraprostatic extension	3
Pathological result of divided surface in excised prostate	
Biopsy specimens in divided surface (samples)	141
Pathology results in divided surface (samples)	
Normal	121
Adenocarcinoma	20
Gleason score 2-6	9
7	6
8-10	5
Diagnostic Accuracy of PDD in divided surface of excised prostate	
Positive fluorescence (samples/rate (%))	31/22.0
Prediction accuracy (%)	48.4
Sensitivity (%)	75.0
Specificity (%)	87.3

## Discussion

The number of new cases of prostate cancer detected by PSA screening is increasing every year; in particular, the number of low-risk cases with an indication for surgery. Radical prostatectomy is the gold standard therapy for prostate cancer in low-risk patients. The purpose of radical prostatectomy is to remove the whole cancerous prostate. However, positive surgical margins after radical prostatectomy were detected in 14–26 % of cases [2–5]. A histologically positive surgical margin is a major risk factor for biochemical recurrence and disease progression after radical prostatectomy. It was shown that positive surgical margins were associated with a 2.6-fold increased unadjusted risk of prostate-cancer-specific mortality [hazard ratio (HR) 2.55, 95 % confidence interval (CI) 2.02–3.21], and remained an independent predictor of prostate-cancer-specific mortality on multivariate analysis (HR 1.70, 95 % CI 1.32–2.18) in 65,633 patients who underwent radical prostatectomy for prostate cancer [20]. Overall, we believe it is important to reduce positive surgical margins for cancer control.

Kriegmair et al. published the first report about the feasibility of ALA-PDD for bladder cancer in 1996 [21]. Subsequently, Hungerhunber et al. reported a greater number of cases and demonstrated the feasibility of ALA-PDD [22]. Zaak et al. reported the first experience of intraoperative ALA-PDD for prostate cancer in 2008 [15], and revealed its feasibility for detecting positive surgical margins. Adam et al. and Ganzer et al. revealed the diagnostic accuracy, with overall sensitivity and specificity of 56–75 % and 88.2–91.6 %, respectively [16, 23]. The present study showed that one case had a red-fluorescence surgical margin of the excised whole prostate and a histologically confirmed positive surgical margin of Gleason score 6. Heat degeneration using an electrical device and the length of the positive surgical margin have a crucial influence on ALA-PDD. Heat degeneration leads to damage of intracellular PpIX, so we could not detect red fluorescence. Besides these factors, we could not detect red fluorescence in a case with a short positive surgical margin because the total amount of PpIX in the tumor cells was small. In a case with an 8.2-mm-long positive surgical margin, we could detect red fluorescence, but not in a case with a 1.5-mm margin. Therefore, it is possible that a positive surgical margin length <3 mm makes it difficult to detect red fluorescence in ALA-PDD. In cases of extraprostatic extension, the tumor cells were not exposed on the surface of the prostate and were covered with prostatic capsule or peripheral fat tissue. The penetration of blue light was low at 0.2 mm [24]. Blue light has difficulty penetrating deeply into tissue and has a direct effect only on the surface of tissue. Therefore, it is difficult to detect red fluorescence on extraprostatic extension. In conclusion, heat degeneration by an electric device and short linear length of positive surgical margin constitute limiting factors for ALA-PDD.

PDD of the divided surface of excised prostate could detect prostate cancer cells with a high degree of accuracy. We have previously shown *in vitro* accumulation of ALA-mediated PpIX in prostate cancer cell lines [25]. In the present study of the divided surface, we could clinically identify red-fluorescence emission in human prostate cancer cells and predominant accumulation of PpIX in cancerous tissue. In contrast, we could not detect red-fluorescence emission in normal prostate tissue. In terms of the diagnostic accuracy of PDD in bladder cancer, the overall sensitivity and specificity were 93.4 % and 58.9 %, respectively [26]. Meanwhile, sensitivity and specificity were 75.0 % and 87.3 %, respectively, in the divided surface of prostate. In terms of the prognostic accuracy of prostate cancer, there were lower sensitivity and higher specificity than for bladder cancer. Thus, the diagnostic accuracy of PDD varies according to the type of cancer. In addition, false-positive findings in prostate cancer were observed in basal hyperplasia with chronic inflammation similar to bladder cancer. Considering the location of prostate cancerogenesis, the capsule of the prostate has little effect on basal hyperplasia and chronic inflammation. Therefore, false-positive findings have no significant effect on intraoperative PDD.

This study showed preliminary results of intraoperative ALA-PDD in radical prostatectomy. We conclude that intraoperative ALA-PDD is feasible, but our study was limited by clinical stage and heat degeneration. In the future, a randomized trial should be carried out.

## Conclusions

Intraoperative ALA-PDD for prostate cancer is helpful in assessing the presence of residual tumor in the surgical margins. Clearly, future randomized studies are needed to examine high-risk prostate cancer patients.

## Abbreviations

ALA: Aminolevulinic acid
PDD: Photodynamic diagnosis
PpIX: Protoporphyrin IX
PSA: Prostate-specific antigen
